# Construction and analysis of heart failure diagnosis model based on random forest and artificial neural network

**DOI:** 10.1097/MD.0000000000031097

**Published:** 2022-10-14

**Authors:** Chen Boyang, Li Yuexing, Yan Yiping, Yu Haiyang, Zhang Xufei, Guan Liancheng, Chen Yunzhi

**Affiliations:** a School of Preclinical Medicine, Guizhou University of Traditional Chinese Medicine, Guiyang, Guizhou, China; b Second Affiliated Hospital, Guizhou University of Traditional Chinese Medicine, Guiyang, Guizhou, China.

**Keywords:** artificial neural network, diagnostic model, heart failure, random forest

## Abstract

Heart failure is a global health problem and the number of sufferers is increasing as the population grows and ages. Existing diagnostic techniques for heart failure have various limitations in the clinical setting and there is a need to develop a new diagnostic model to complement the existing diagnostic methods. In recent years, with the development and improvement of gene sequencing technology, more genes associated with heart failure have been identified. We screened for differentially expressed genes in heart failure using available gene expression data from the Gene Expression Omnibus database and identified 6 important genes by a random forest classifier (ASPN, MXRA5, LUM, GLUL, CNN1, and SERPINA3). And we have successfully constructed a new heart failure diagnostic model using an artificial neural network and validated its diagnostic efficacy in a public dataset. We calculated heart failure-related differentially expressed genes and obtained 24 candidate genes by random forest classification, and selected the top 6 genes as important genes for subsequent analysis. The prediction weights of the genes of interest were determined by the neural network model and the model scores were evaluated in 2 independent sample datasets (GSE16499 and GSE57338 datasets). Since the weights of RNA-seq predictions for constructing neural network models were theoretically more suitable for disease classification of RNA-seq data, the GSE57338 dataset had the best performance in the validation results. The diagnostic model derived from our study can be of clinical value in determining the likelihood of HF occurring through cardiac biopsy. In the meantime, we need to further investigate the accuracy of the diagnostic model based on the results of our study.

## 1. Introduction

Heart failure (HF) is a clinical syndrome in which structural and functional defects of the myocardium lead to impaired ventricular filling and/or ejection.^[1]^ Depending on the functional status of the heart, HF is classified as heart failure with preserved ejection fraction (HFpEF) and heart failure with reduced ejection fraction (HFrEF). HF with mid-range ejection fraction is controversial due to its nature and was not included in our current study. And the mechanisms of development of these two types of HF are different. The prevalence of HFpEF accounts for over 50% of all HF cases and this figure is expected to rise further.^[2]^ However, most of the available drug therapies focused on HFrEF have shown little effect in treating patients with HFpEF.^[3]^ We therefore need new therapies that will help treat HFpEF and ultimately improve the quality of life and health status of these HF patients.

**Figure 1.**
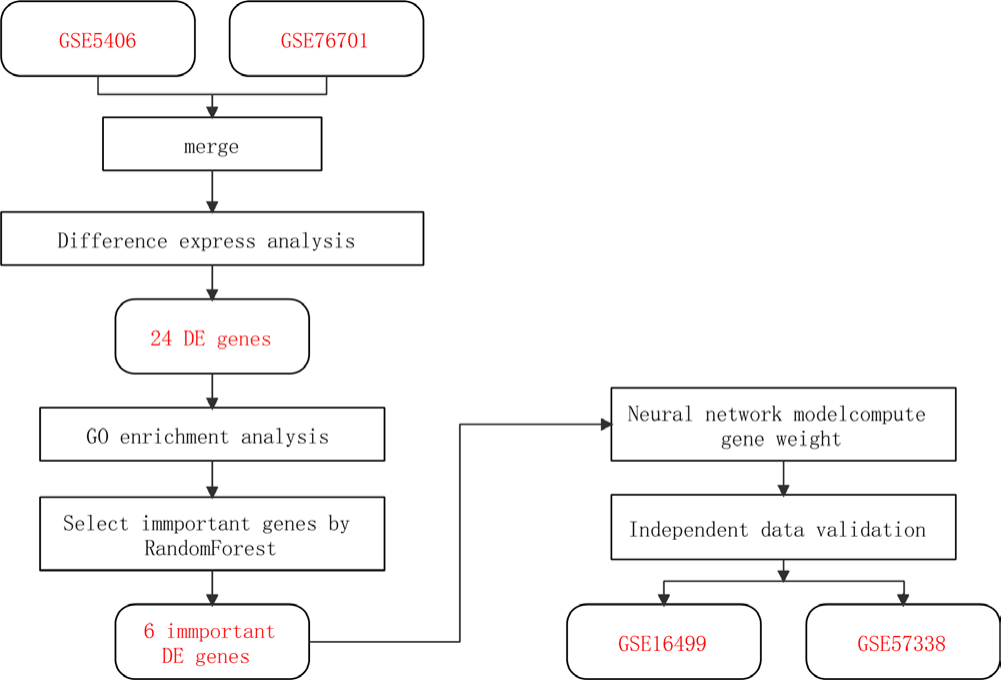
Flowchart.

There are limitations to the diagnostic techniques commonly used in clinical practice for HF. For example, brain natriuretic peptide/N-terminal-proB-type natriuretic peptide levels may also be elevated in non-HF diseases such as pulmonary hypertension, acute or chronic renal failure, and cirrhotic ascites, but are normal in patients with HFpEF.^[4,5]^ Echocardiography is a technical device commonly used in clinical practice to measure cardiac function, and relies mostly on individual operation proficiency and diagnostic experience of specialists, making the results less reproducible. In addition, it is difficult to determine patients with HFpEF by measuring the ejection fraction value.^[6]^ Hence, there is a need to develop new diagnostic models to complement these existing methods. In recent years, the rapid development of second-generation sequencing technologies has helped to identify a wide range of disease-associated marker genes, providing the technical basis for the development of new gene-related diagnostic models for HF. In the present study, we screened differentially expressed genes (DEGs) between HF and normal myocardial samples according to the Gene Expression Omnibus database (GEO). On the basis of these DEG data, we used the random forest algorithm to identify the key genes expressed in HF. Next, we construct a genetic diagnostic model of HF by feeding these key genes into an artificial neural network (Fig. 1).

## 2. Materials and Methods

### 2.1. Data download and processing

GEO database^[7]^ was used for downloading data to obtain the expression profile and clinical phenotype data of microarray datasets GSE5406 and GSE76701 and RNA-seq datasets GSE16499 and GSE57338, and the information of the chip probes of the corresponding platforms. During the conversion of microarray probe IDs and gene symbols, multiple probes were found to correspond to 1 gene symbol. In this case, the average probe expression was used as the gene expression level. The Perl (version 5.30.2.1) was used to perform gene ID conversion on the RNA-seq expression profile.

### 2.2. Differential expression and enrichment analysis

Normal and HF sample genes from the GSE5406 and GSE76701 datasets were combined using the R packages “limma” and “sva,” and only one duplicate gene was taken and then conducted differential analysis. The limma software package^[8]^ uses the classic Bayesian data analysis to screen DEGs. The significance criteria for DEGs were set at a *P* value of <.05 and logFoldChang (logFC) >1.0. The pheatmap software package was used to draw the heat map of DEGs, and the R package clusterProfiler^[9]^ was used to perform GO function enrichment analysis on related genes to identify 3 types of significantly enriched GO terms (*P* < .05).

### 2.3. Random forest screening for important genes

The randomForest package^[10]^ was used to construct a random forest model of DEGs. First, the average model miscalculation rate of all genes based on out-of-band data was calculated. 500 was chosen as the best number of trees contained in the random forest. Next, a random forest model was constructed and the genes with an importance value >2 were chosen as the disease specific genes for the subsequent model construction. The software package pheatmap was used to reclassify the unsupervised hierarchical clusters of the important genes in the GSE5406 and GSE76701 dataset and draw a heat map.

### 2.4. Neural network to build disease classification model

Genes with importance scores >2 in the previous step were selected for neural network model training. After the data was normalized to the maximum and minimum values, the R software package neuralnet (version 1.44.2) was used to construct an artificial neural network model of the important variables. Five hidden layers were set as the model parameters to construct a classification model of HF diseases through the obtained gene weight information. In this model, the sum of the product of the weight scores multiplied by the expression levels of the important genes was used as the disease classification score. The p receiver-operating characteristic software package^[11]^ was used to calculate the verification results of area under curve (AUC) classification performance.

### 2.5. Additional data verification

The classification score model for the constructed HF diseases and the normal samples was tested for effectiveness verification on 2 independent datasets (GSE16499 and GSE57338). The pROC software package was used to draw the ROC curves for each dataset, and the area under the ROC curve was calculated to verify the classification efficiency.

## 3. Results

### 3.1. Differential expression analysis

**Figure 2.**
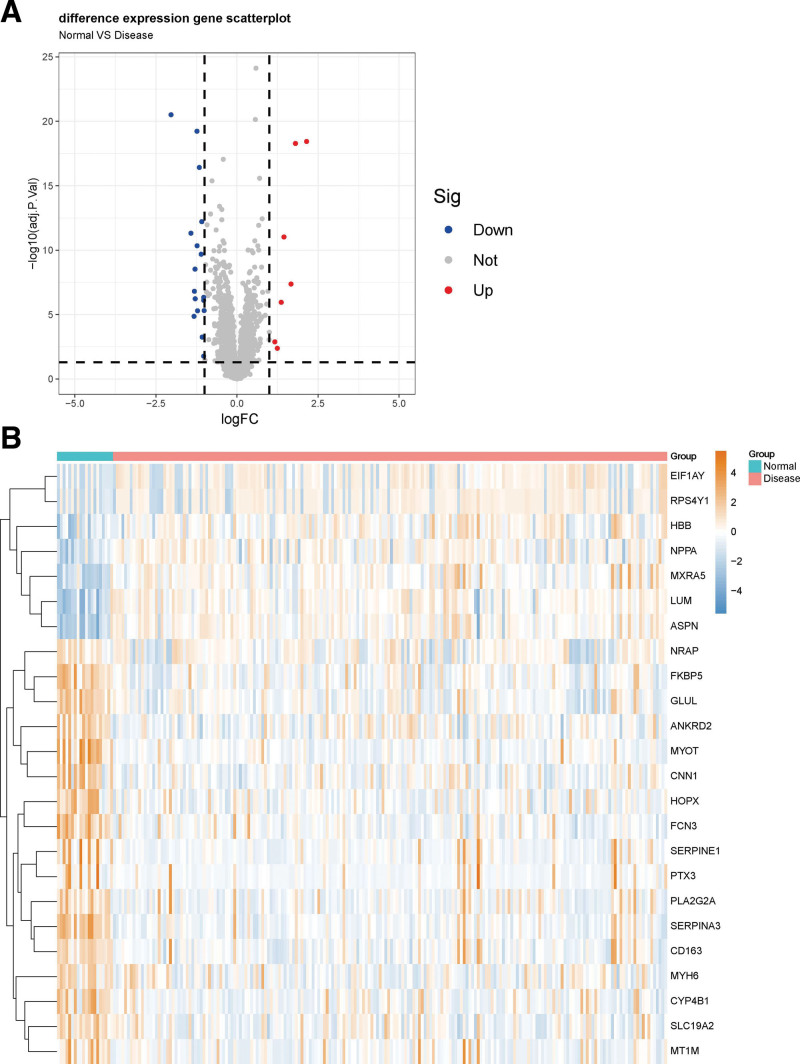
(A) Volcano plot of differential expression analysis results. The abscissa is logFC and the ordinate is -log10 *P* value. The upper right part has a *P* value <.05 and a fold change greater than 1.0, indicating significant DEGs with higher expression levels. The upper left part has a *P* value <.05 and a fold change <−1.0, indicating significant DEGs with reduced expression. The gray dots represent the remaining stable genes. (B) Heatmap of DEGs. The colors in the graph from dark orange to steel blue indicate high to low expression. On the upper part of the heatmap, the red band indicates the disease samples and the blue band indicates the normal samples. DEG = differentially expressed genes.

Differential expression analysis was performed based on the chip datasets GSE5406 and GSE76701 to screen for DEGs. The GSE5406 dataset contained 210 samples, including 16 normal and 194 HF disease samples and the GSE76701 dataset contained 8 samples, including 4 normal and 4 HF disease samples. Next, the sva package was used to merge the normal samples and the HF samples of the 2 datasets. Then, the limma package was used to identify DEGs between the HF samples of this chip dataset and the normal control samples through the Bayesian test. The results of the DEGs are shown in the volcano graph (Fig. 2A) and heatmap (Fig. 2B). Based on fold change values of > 1.0 and significance threshold of *P* < .05, we identified 24 significant DEGs related to HF diseases by the screen.

### 3.2. GO enrichment analysis

**Figure 3.**
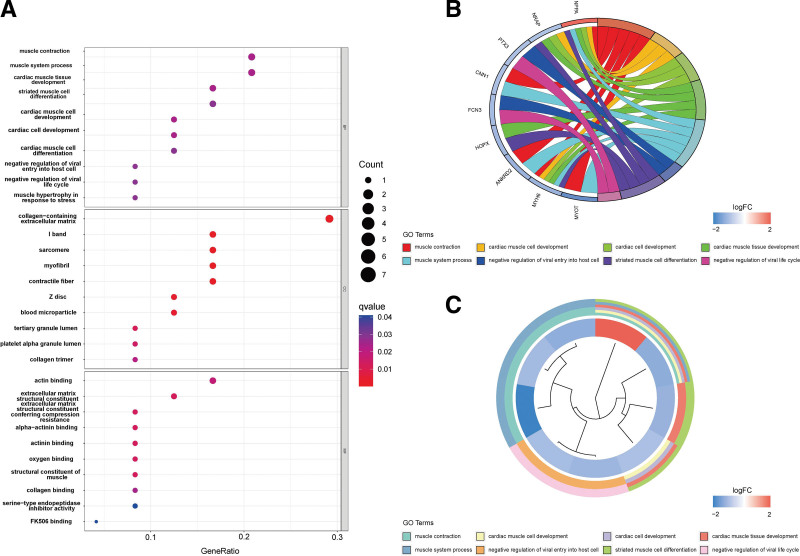
Graph showing the enrichment analysis results. (A) Bubble plot of GO enrichment results. Biological processes are shown on the top, cellular components are shown in the middle, and molecular function is shown on the down. A bubble represents a GO term, with the size of the bubble indicating the number of genes in the GO term. (B) Ring plot showing GO enrichment. The left side indicates the DEGs, the red gene band indicates upregulation, and blue indicates downregulation. The band on the right with different colors represents different GO terms. The connecting line indicates that the gene is included in the GO term. (C) Clustering diagram showing GO enrichment. The inside indicates the DEGs, the red gene band indicates upregulation, and blue indicates downregulation. The band outside with different colors represents different GO terms. DEG = differentially expressed genes.

Gene ontology (GO) enrichment analysis was performed on the 24 significant DEGs using the clusterProfiler package. The Benjamini–Hochberg correction method was used, with the thresholds set at a *P* value of < .05 and a *Q* value of < 0.05. Figure 3A shows the analysis results of 3 aspects of GO enrichment, including biological processes, cellular components, and molecular function. Among the results, the related biological processes involved in HF include muscle contraction, muscle system process and cardiac muscle tissue development. The cellular components involved include collagen-containing extracellular matrix (ECM). The molecular functions included actin binding. Figure 3B and 3C show part of the GO enriched terms and the significant DEGs involved.

### 3.3. Random forest screening for DEGs

**Figure 4.**
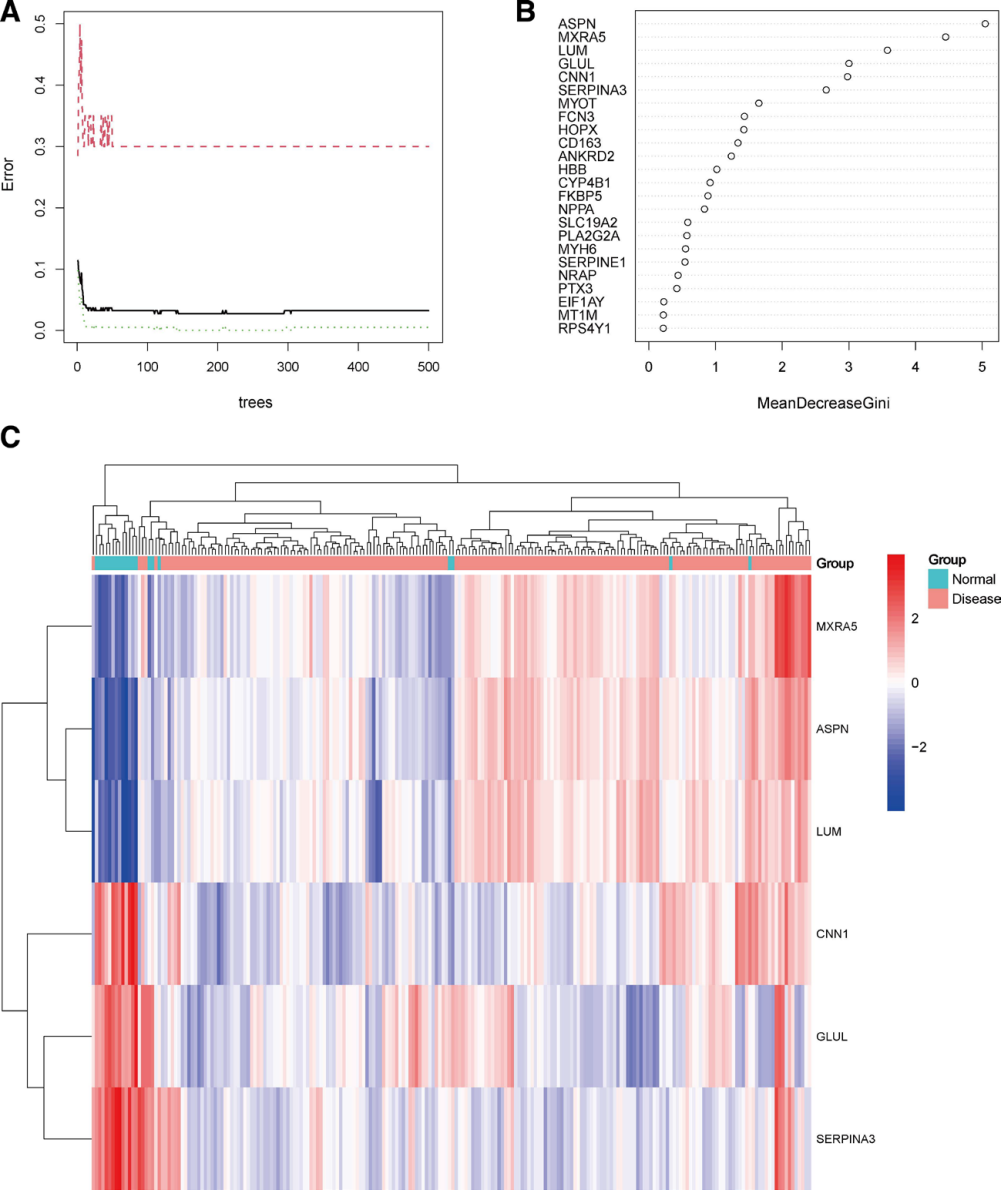
(A) The influence of the number of decision trees on the error rate. The x-axis represents the number of decision trees, and the y-axis indicates the error rate. When the number of decision trees is approximately 330, the error rate is relatively stable. (B) Results of the Gini coefficient method in random forest classifier. The x-axis indicates the genetic variable, and the y-axis represents the importance index. (C) Heatmap of unsupervised clustering showing the results of the hierarchical clustering produced by the 6 important genes generated by random forest in GSE5406 and GSE76701 datasets. Red color indicates genes with high expression in the samples, blue color indicates genes with low expression in the samples, the blue band on the upper side of the heatmap indicates normal samples, and the red band indicates HF disease samples. HF = heart failure.

Next, we input the 24 DEGs into the random forest classifier. Finally, we chose 6 as the parameter of variable number. The number of variables was as small as possible, and the out-of-band error was as low as possible. Referring to the relationship plot between the model error and the number of decision trees (Fig. 4A), we selected 500 trees as the parameter of the final model, which showed a stable error in the model. We then identified 6 DEGs with an importance >2 as the candidate genes for subsequent analysis. Figure 4B shows that among the 6 variables, ASPN and MXRA5 were the most important, followed by LUM, GLUL, CNN1, and SERPINA3. Based on these 6 important variables, we performed k-means unsupervised clustering of the merge of the GSE5406 and GSE76701 datasets. Figure 4C shows that the 6 genes could be used to distinguish between the disease and normal samples in 218 samples of the dataset. Among them, ASPN, MXRA5 and LUM genes are a cluster with low expression in the normal samples and high expression in the disease samples. On the other hand, GLUL, CNN1, and SERPINA3 belong to another cluster with high expression in the normal samples and low expression in the disease samples.

### 3.4. Construction of the artificial neural network model

Genes with importance scores >2 were selected to be trained as neural network models. The maximum and minimum data values were standardized and the number of hidden layers was set as 5. In the choice of parameters, there was no fixed rule on how many layers and neurons were to be used. The number of neurons should be between the input layer size and the output layer size, usually two-thirds of the input size. Thus, the parameter of number of neurons was set as 6. The dataset was randomly divided into a training set and a verification set. The purpose of the training set was to determine the weights of candidate DEGs. The verification set was used to verify the classification efficiency of the model score constructed with gene expression and gene weight.

**Figure 5.**
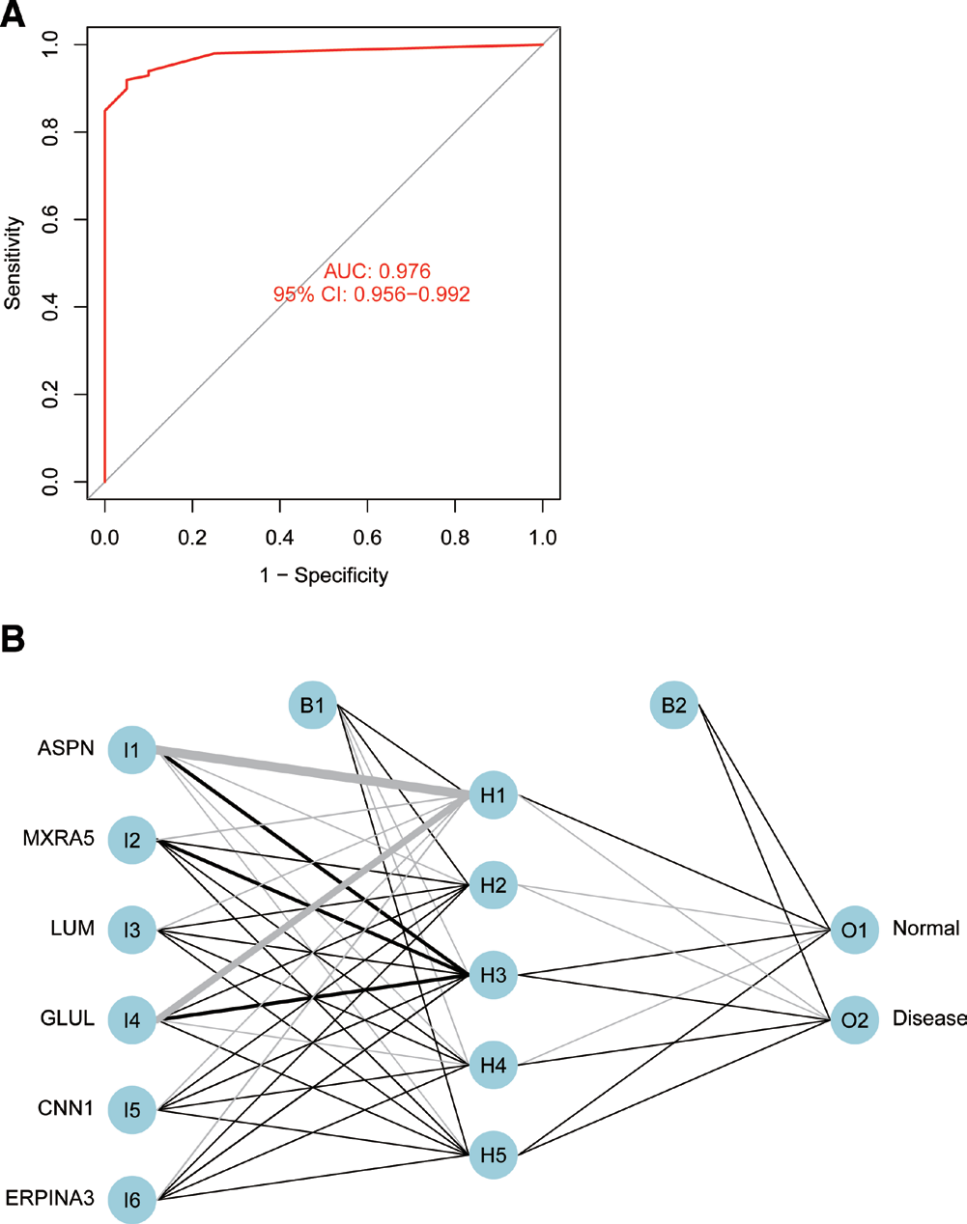
(A) Verification of the ROC curve results. (B) Results of neural network visualization. ROC = receiver-operating characteristic.

The results display the model classification performance using the ROC curve (Fig. 5A). The areas under the ROC curves (AUC) of the results were close to 1 (AUC = 0.976), which shows the robustness of the model. Therefore, we next used all the data to construct the neural network model (Fig. 5B).

### 3.5. Evaluation of AUC

**Figure 6.**
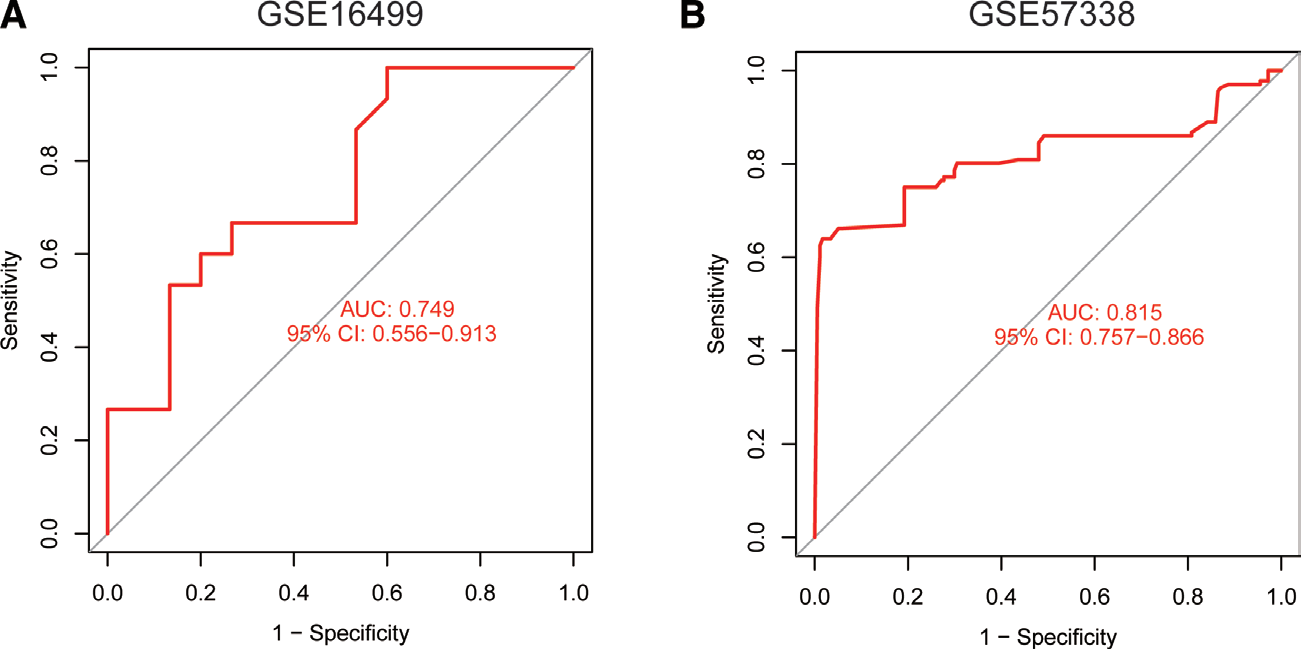
Plot showing AUC verification results. (A) AUC verification results in the GSE16499 dataset. (B) AUC verification results in the GSE57338 dataset. The AUC value is the area under the ROC curve. AUC = area under the curve, ROC = receiver-operating characteristic.

Using the 3 independent verification datasets of GSE16499 and GSE57338, the 2 scores were calculated and their classification efficiency was evaluated, and the AUC were compared. Figure 6 shows a comparison of the 3 scores of the 2 independent verification datasets. In the GSE16499 dataset (Fig. 6A), the AUC was 0.749. In the GSE57338 dataset (Fig. 6B), the AUC was 0.815.

## 4. Discussion

In this study, we calculated HF-related DEGs and obtained 24 candidate genes by random forest classification, and selected the top 6 genes as important genes for subsequent analysis. The prediction weights of the genes of interest were determined by the neural network model and the model scores were evaluated in 2 independent sample datasets. Since the weights of RNA-seq predictions for constructing neural network models were theoretically more suitable for disease classification of RNA-seq data, the GSE57338 dataset had the best performance in the validation results. However, due to the lack of genetic data of HFpEF in the GEO database, the genetic characteristics of HFpEF were not included in the diagnostic model, thus affecting the diagnostic validity of the HFpEF model.

Asporin (ASPN) is a secreted matrix protein associated with cancer, osteoarthritis and periodontal membrane mineralization. The transforming growth factor-β (TGF-β) superfamily plays multiple roles in cell differentiation, proliferation and fibrosis. Overexpression of TGF-β1 in the myocardium induces myocardial hypertrophy, which eventually progresses to HF.^[12]^ In addition, TGF-β1 and its downstream typical Smad signaling play an important role in the process of cardiac fibrosis.^[13]^ ASPN promotes the migration and invasion of colorectal cancer cells through the TGF-β/Smad2/3 pathway and can be used as a potential prognostic biomarker for patients with colorectal cancer.^[14]^ ASPN has been less studied in HF, but one study shows that ASPN expression is upregulated in cardiac fibroblasts from HF patients as well as HF mice, which was consistent with our analysis.^[15]^ ASPN may be involved in HF by downregulating Bcl-2, upregulating TGF-1, Bax, type III collagen, and fibronectin, and phosphorylating Smad2 and Smad3 to increase apoptosis in H9C2 cardiomyocytes.^[16]^ However, a recent study has shown that ASPN can prevent adverse cardiac remodeling and prevent excessive cardiac fibrosis and cardiomyocyte death.^[17]^ Although ASPN plays a cancer-promoting role as an oncogene, studies in HF are still limited, but this also provides us with a basis for better studies in the future. Matrix remodeling-associated protein 5 (MXRA5) belongs to the MXRA gene family, an adhesion protein with a leucine-rich repeat sequence and a nucleotide-associated (Adlican) immunoglobulin structural domain, expressed in primates but not in rats and mice, and plays an important role in cell adhesion and matrix remodeling.^[18]^ The expression of MXRA5 mRNA has been observed to be upregulated in cardiac tissue in ischemic cardiomyopathy, and may play an important role in the development and progression of HF.^[19]^ The MAPK pathway plays a key role in the regulation of cell proliferation, survival, differentiation and apoptosis, and its activation promotes myocardial fibrosis and reduces cardiac function in HF rats.^[20]^ It has been shown that MXRA5 is closely related to the MAPK pathway, and that MXRA5 promotes cell proliferation and apoptosis through the MAPK pathway.^[21]^ Therefore, it is speculated that MXRA5 may play an important role in HF through the MAPK pathway. Furthermore, MXRA5 is a new ECM biomarker for calcified aortic valves, and its increased expression level leads to aortic stenosis, which in turn induces the development of HF.^[22]^ Lumican (LUM) is a proteoglycan secreted by the ECM that regulates collagen fibril formation and is highly expressed in HF patients and mice. NF-κB-mediated inflammation induces LUM production by cardiac fibroblasts, and increased LUM promotes raised levels of TGF-β1, exacerbates fibrosis in cardiac fibroblasts and promotes cell adhesion through the TGF-β2/Smad 2 signaling cascade promoting cell adhesion, which in turn exacerbates HF.^[23,24]^ The osteopontin is an effector of extracellular signals that induces and maintains the growth and fibrosis of cardiomyocytes. Treatment with osteopontin inducers at the time of surgery blocks osteopontin downstream signaling (PI3K and Akt phosphorylation), reduces LUM gene expression, prevents cardiomyocyte hypertrophy and cardiac fibrosis, and improves cardiac dysfunction.^[25]^ Besides, LUM also plays a key role in innate immunity, which may have important implications for HF treatment.^[26]^ Calponin 1 (CNN1) is an actin filament-associated regulatory protein that is specifically expressed in smooth muscle cells and functions to regulate contractile actin–myosin filaments and the noncontractile actin cytoskeleton in smooth muscle cells, and plays a role in fine-tuning smooth muscle contractility.^[27]^ A study on gene expression fingerprinting in human HF shows that CNN1 expression is downregulated in hearts with HF,^[28]^ which is generally consistent with the results of the present study. The ERK1/2 signaling pathway is involved in angiotensin II-induced G protein signaling to induce activation of the CNN1 gene.^[29]^ Activation of ERK1/2 has been shown to be cardioprotective against ischemia-reperfusion injury in vivo.^[30]^ CNN1 improves the dilated cardiomyopathy phenotype in mice via the ERK1/2 pathway, suggesting that CNN1 may be a therapeutic target for controlling the development of dilated cardiomyopathy and HF.^[31]^ The reduction of CNN1 levels caused by oxidative stress has also been shown to promote hypertrophic arterial remodeling, possibly contributing to the development of HF.^[32]^ Glutamate-ammonia ligase (GLUL), also known as glutamine synthetase, is an enzyme that catalyzes the synthesis of glutamine from glutamate and ammonia in an ATP-dependent reaction.^[33]^ This protein plays a role in ammonia and glutamate detoxification, acid-base homeostasis, cell signaling, and cell proliferation.^[34]^ Endothelial function plays an important role in HF, with endothelial cell metabolism controlling angiogenesis.^[35]^ Gene deletion of GLUL in endothelial cells impairs angiogenesis, while pharmacological blockade of glutamine synthetase also inhibits angiogenesis in ocular and inflammatory skin diseases, and GLUL knockdown induces actin stress fibers and impedes endothelial cell motility.^[36]^ GLUL may therefore have a regulatory role in endothelial dysfunction in HF and could be used as a therapeutic target. SERPINA3 (Serpin peptidase inhibitor clade A member 3), also known as a1-antichymotrypsin, is a serine protease inhibitor involved in a wide range of biological processes.^[37]^ It is involved in the regulation of immune cells mainly through the regulation of cathepsin G and elastase.^[38]^ Cathepsin G is found mainly in neutrophils and is released during inflammation and activates the body’s immune response.^[39]^ However, prolonged activation of neutrophil accumulation leads to indirect damage to the myocardium and therefore inhibition of neutrophil accumulation may reduce the incidence of HF.^[40]^ A proteomic analysis shows that SERPINA3 expression levels are significantly increased in epicardial adipose tissue of HF patients and positively correlated with brain natriuretic peptide levels.^[41]^ Moreover, the heart tissue of HF patients can itself secrete SERPINA3, which induces the growth and carcinogenesis of colon cells.^[42]^ Patients with new or worsening HF were more likely to die or be readmitted to the hospital with elevated SERPINA3 levels.^[43]^ Therefore, SERPINA3 may become an important prognostic marker for HF patients in the future.

In summary, our current study only adds to the existing diagnostic and therapeutic approaches. However, the current diagnostic criteria and procedures are based on data from patients with HFrEF and their applicability to patients with HFpEF is unclear, and it is not possible to judge the severity of HFpEF. Therefore, the diagnostic model derived from our study can be of clinical value in determining the likelihood of HF occurring through cardiac biopsy. In the meantime, we need to further investigate the accuracy of the diagnostic model based on the results of our study.

## Acknowledgments

We thank the authors who provided the GEO public datasets.

## Author contributions

Chen Boyang designed research, analyzed data and wrote the paper that was revised by Chen Yunzhi. All authors have read and approved the final version of the manuscript.

Formal analysis: Li Yuexing, Yan Yiping.

Data curation: Yu Haiyang, Zhang Xufei, Guan Liancheng.

Project administration: Chen Yunzhi.

**Conceptualization:** Chen Boyang.

**Data curation:** Chen Boyang, Guan Liancheng, Yu Haiyang, Zhang Xufei.

**Formal analysis:** Chen Boyang, Li Yuexing, Yan Yiping.

**Project administration:** Chen Yunzhi.
